# Inflammatory responses driven by neutrophil extracellular traps in cardiovascular diseases: molecular mechanisms, emerging biomarkers, and therapeutic targets

**DOI:** 10.3389/fimmu.2026.1879572

**Published:** 2026-07-29

**Authors:** Aolong Wang, Jingjing Wei, Rui Yu, Ming Li, Bin Li, Xinlu Wang, Mingjun Zhu

**Affiliations:** 1 Department of Cardiovascular Disease, The First Affiliated Hospital of Henan University of Chinese Medicine, Zhengzhou, China; 2 The First Clinical Medicine College, Henan University of Chinese Medicine, Zhengzhou, China; 3 Collaborative Innovation Center of Prevention and Treatment of Major Diseases by Chinese and Western Medicine, Zhengzhou, China; 4 Henan Evidence-based Medicine Center of Chinese Medicine, The First Affiliated Hospital of Henan University of Chinese Medicine, Zhengzhou, China

**Keywords:** biomarkers, cardiovascular diseases, chronic inflammation, neutrophil extracellular traps, therapeutic targets

## Abstract

Cardiovascular disease (CVD) remains the leading cause of mortality and disability worldwide, characterized by high incidence, morbidity, and mortality. Although guideline-directed medical therapy has improved clinical outcomes and slowed disease progression to some extent, a substantial residual risk persists, and the progression of CVD has not been fundamentally halted. Beyond traditional risk factors such as hypertension, diabetes mellitus, and dyslipidemia, dysregulated inflammatory responses are now recognized as a central driver of CVD initiation, progression, and adverse outcomes. Neutrophil extracellular traps (NETs), as key effectors of innate immunity, contribute to inflammatory cascades through mechanisms including inflammasome activation, modulation of immune responses, and promotion of thrombosis. Accumulating evidence indicates that NETs play a critical role in a spectrum of cardiovascular conditions, including atherosclerosis, myocardial infarction, and heart failure, and are closely associated with poor prognosis. This review systematically summarizes the mechanisms by which NETs mediate inflammatory responses in CVD and discusses their roles across different disease contexts. It further explores the potential of NETs as inflammation-related biomarkers and therapeutic targets, with the aim of providing new insights into inflammation-based strategies for the prevention and treatment of CVD.

## 1 Introduction

Cardiovascular disease (CVD) remains the leading cause of death and disability worldwide, characterized by high incidence, mortality, and substantial economic burden. It is estimated that approximately 626 million individuals are affected by CVD globally (1), with nearly 19 million deaths each year, accounting for about 32% of all-cause mortality (2). With ongoing population aging, the global burden of CVD continues to rise. In recent years, guideline-directed medical therapy has significantly reduced the incidence and mortality of CVD. However, considerable residual risk persists, and long-term outcomes remain suboptimal. A growing body of evidence highlights the pivotal role of inflammation in the initiation, progression, and adverse outcomes of CVD (3–5). Beyond traditional risk factors such as hypertension, diabetes mellitus, and dyslipidemia, inflammation is now recognized as a central driver of disease progression (5, 6). It contributes to pathogenesis through multiple mechanisms, including endothelial dysfunction, lipid accumulation, myocardial fibrosis, immune cell recruitment, and increased plaque instability (7, 8). Clinical studies further demonstrate that targeting inflammatory pathways can reduce the risk of major adverse cardiovascular events (MACE) (9). Therefore, elucidating inflammation-related mechanisms and identifying novel biomarkers and therapeutic targets are essential for optimizing CVD prevention and treatment strategies.

Aberrant activation of innate immunity is a key driver of inflammatory amplification and persistence. Neutrophil extracellular traps (NETs), as critical mediators linking innate immunity and inflammatory responses (10), are web like chromatin structures released by activated neutrophils. They consist primarily of a DNA-histone backbone decorated with bioactive components such as neutrophil elastase (NE) and myeloperoxidase (MPO). Acting as damage-associated molecular patterns (DAMPs), NETs can amplify immune inflammatory responses, thereby inducing endothelial injury, amplifying inflammatory cascades, and promoting thrombosis (11–13). Emerging evidence indicates that NETs are widely involved in inflammatory processes associated with CVD, including plaque destabilization in atherosclerosis (AS), myocardial infarction (MI), ischemia reperfusion (I/R) injury, heart failure (HF), and microvascular dysfunction (14). Their levels are closely associated with disease onset, progression, and adverse prognosis (15), suggesting their potential as novel biomarkers and therapeutic targets in CVD. Nevertheless, the mechanisms by which NETs contribute to cardiovascular inflammation have not been comprehensively summarized. Further elucidation of their roles in inflammatory responses will deepen our understanding of disease pathophysiology and provide a theoretical basis for biomarker development and targeted interventions.

## 2 Definition and formation mechanisms of NETs

### 2.1 Definition of NETs

NETs are a key effector mechanism of innate immunity, representing specialized structures released by activated neutrophils. They are primarily composed of decondensed chromatin formed by DNA and histone complexes and are decorated with various granular proteins, including NE, MPO, and cathepsin G (CG), while histones represent nearly 70% of the proteins associated with NETs (16). Due to their unique structural properties, NETs effectively trap and eliminate pathogens such as bacteria, fungi, and viruses, thereby playing a critical role in host defense. The formation of NETs, termed “NETosis,” is a distinct form of programmed cell death that differs from classical apoptosis and necrosis (17). Upon stimulation by microbial pathogens, inflammatory mediators, or other endogenous and exogenous factors, intracellular reactive oxygen species (ROS) generation increases, leading to activation of peptidyl arginine deiminase 4 (PAD4). PAD4 catalyzes histone citrullination, promoting chromatin decondensation and nuclear envelope disintegration (18). The decondensed chromatin subsequently associates with granular proteins such as NE and MPO and is released into the extracellular space, ultimately forming the functional NET structure.

### 2.2 Types and formation mechanism of NETs

**Figure 1:**
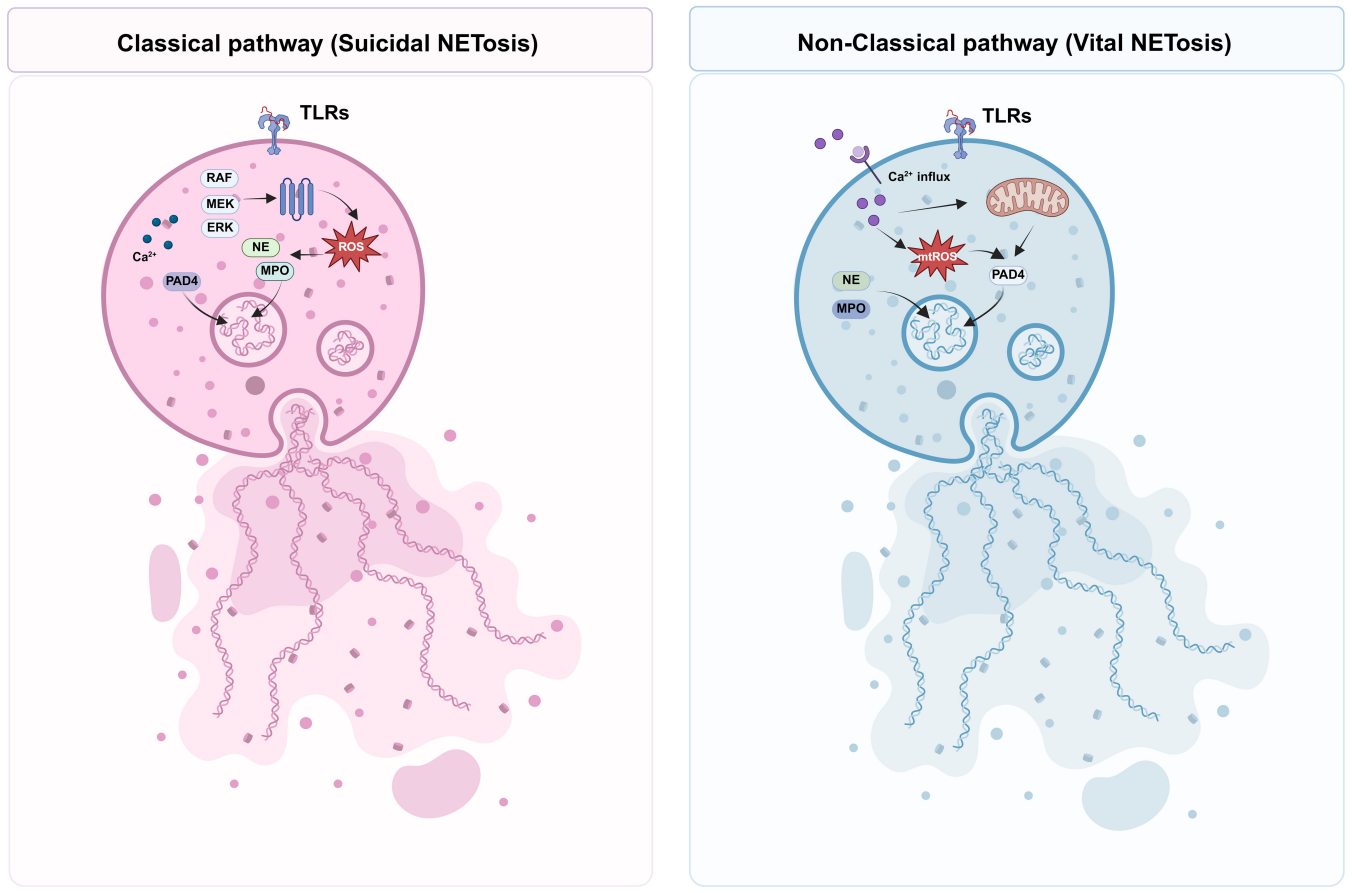
Classical and non-classical pathways of neutrophil extracellular trap (NET) formation. Classical NETosis is triggered by extracellular stimuli such as bacteria and lipopolysaccharide (LPS), leading to activation of the mitogen-activated protein kinase (MAPK)/protein kinase C (PKC) and Raf (rapidly accelerated fibrosarcoma)-MEK (MAPK/ERK kinase)-ERK (extracellular signal-regulated kinase) signaling pathways, followed by nicotinamide adenine dinucleotide phosphate (NADPH) oxidase-derived reactive oxygen species (ROS) production. ROS activate neutrophil elastase (NE) and myeloperoxidase (MPO), while peptidylarginine deiminase 4 (PAD4)-mediated histone citrullination promotes chromatin decondensation. Caspase-4/5/11-mediated gasdermin D (GSDMD) cleavage further induces plasma membrane rupture, resulting in extracellular release of chromatin-protein complexes and neutrophil death. Non-classical NETosis occurs without plasma membrane rupture. Extracellular Ca²^+^ influx induces mitochondrial reactive oxygen species (mtROS) production and PAD4 activation, promoting chromatin decondensation. DNA-protein complexes are subsequently released through vesicle-mediated exocytosis, generating anuclear neutrophils that retain partial inflammatory functions.

NET formation can be broadly classified into classical and non-classical pathways, with some studies proposing a mitochondrial-related mechanism (19). The classical pathway, also referred to as suicidal NETosis, is the predominant mode of NET formation and is characterized by neutrophil death following NET release. In response to extracellular stimuli such as bacteria and lipopolysaccharide (LPS), intracellular signaling pathways including mitogen-activated protein kinases and protein kinase C are activated. These signals propagate through the Raf-MEK-ERK cascade, leading to activation of NADPH oxidase (NOX) and subsequent ROS generation, which in turn activates NE and MPO (20). Concurrently, Ca^2+^ dependent PAD4 in the nucleus mediates histone citrullination, further promoting chromatin decondensation (21). In addition, LPS can activate caspase-4/5 or caspase-11, resulting in cleavage of gasdermin D (GSDMD), pore formation in the plasma membrane, and eventual membrane rupture (22). Ultimately, decondensed chromatin complexed with granular proteins is released extracellularly as NETs, accompanied by neutrophil death. In contrast, the non-classical pathway is characterized by the extrusion of chromatin without compromising plasma membrane integrity. In this process, chromatin is packaged into vesicles and exported extracellularly, after which the plasma membrane reseals, giving rise to anucleate neutrophil cytoplasts that retain certain functional capacities (23). This pathway is generally independent of NOX activity and instead relies on extracellular Ca^2+^ influx. Elevated intracellular Ca^2+^ promotes mitochondrial ROS (mtROS) production (24), which activates PAD4 and facilitates its translocation to the nucleus, where it induces histone citrullination and chromatin decondensation. The resulting DNA protein complexes are packaged into vesicles and released via vesicle membrane fusion (20), which depends on ATP generated through glycolysis, and the resulting anucleate neutrophils can continue to participate in the regulation of inflammatory responses (**Figure 1**).

### 2.3 Literature search strategy

This narrative review primarily reviewed English language articles published in PubMed and Web of Science over the past 15 years, with particular emphasis on studies published between 2021 and 2026. The search strategy used combinations of keywords related to NETs, cardiovascular diseases, atherosclerosis, myocardial infarction, ischemia reperfusion injury, heart failure, hypertension, atrial fibrillation, myocarditis, inflammation, biomarkers, and therapeutic targets. Retrieved records were screened by title and abstract to assess their relevance to the role of NETs in cardiovascular inflammation. Full text articles were subsequently evaluated, with priority given to original mechanistic studies, translational investigations, animal experiments, and representative clinical cohort studies that provided mechanistic and pathological insights into the role of NETs in CVD. Studies with limited relevance, insufficient mechanistic evidence, redundant findings, or inadequate methodological reporting were excluded from detailed discussion. In addition, recent high-quality review articles were consulted to provide broader conceptual context and facilitate the interpretation of primary studies.

## 3 Inflammatory response mediated by NETs

Neutrophils are central components of innate immunity, and alterations such as excessive infiltration, sustained activation, functional imbalance, and prolonged survival can collectively drive the amplification of inflammatory responses, thereby contributing to the pathogenesis of various diseases (25, 26). NETs were initially characterized as an essential antimicrobial defense mechanism, capturing and eliminating invading pathogens. However, accumulating evidence indicates that NETs exert context-dependent, dual biological effects. While controlled NET formation contributes to host defense and immune regulation, excessive or dysregulated NET release exhibits pronounced pro-inflammatory activity, largely determined by their composition and post-translational modifications (27). Aberrant NET formation acts as a potent pro-inflammatory stimulus, promoting inflammatory amplification, endothelial injury, and thrombosis. These effects are closely associated with the degree of chromatin decondensation, histone citrullination, and the synergistic interactions between citrullinated histones and DNA. Mechanistically, NETs enhance inflammation through inflammasome activation (28), interactions with DAMPs, and modulation of immune cell responses (29, 30), and have been implicated in a wide spectrum of inflammatory diseases, including respiratory diseases, autoimmune disorders, cardiovascular diseases, and cancer (31–34).

**Figure 2:**
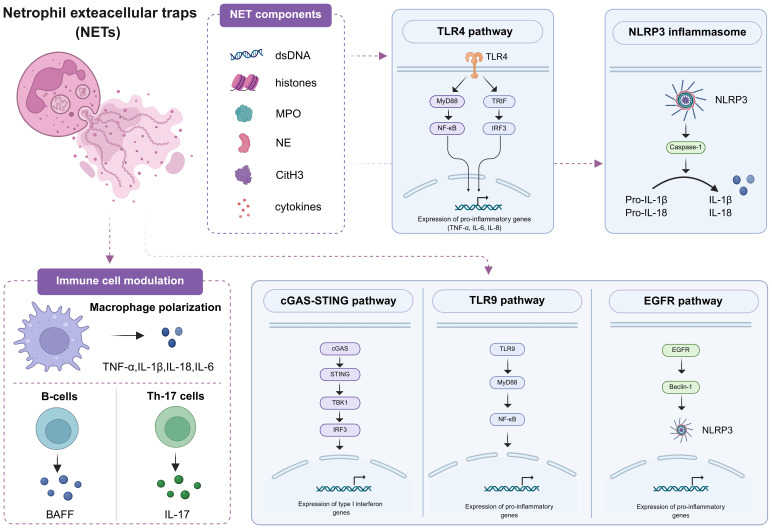
Pro-inflammatory mechanisms of NETs in cardiovascular diseases. NETs serve as an important source and amplification platform of damage-associated molecular patterns (DAMPs). NET-derived double-stranded DNA (dsDNA), histones, MPO, and NE activate pattern recognition receptors, particularly the Toll-like receptor 4 (TLR4) pathway, thereby promoting inflammatory responses. NETs also activate the nucleotide-binding oligomerization domain-like receptor family pyrin domain-containing 3 (NLRP3) inflammasome and caspase-1, inducing interleukin-1β (IL-1β) and interleukin-18 (IL-18) maturation and establishing a positive feedback loop that further enhances NET release. In addition, NETs amplify inflammation through immune cell crosstalk by activating macrophages, neutrophils, and B cells via the cyclic GMP-AMP synthase-stimulator of interferon genes (cGAS-STING), Toll-like receptor 9 (TLR9), and epidermal growth factor receptor-Beclin-1 (EGFR-Beclin-1) pathways, ultimately sustaining inflammatory amplification and cardiovascular injury.

As a major source and amplification platform of DAMPs, NETs release bioactive components such as double-stranded DNA (dsDNA), histones, MPO, and NE, which activate pattern recognition receptors and trigger inflammatory signaling. For instance, histones and dsDNA can activate the Toll-like receptor 4 (TLR4) pathway, thereby promoting inflammation mediated by NETs (30, 35, 36). In parallel, NETs activate the NLRP3 inflammasome, leading to caspase-1 activation and subsequent maturation and release of interleukin-1β (IL-1β) and interleukin-18 (IL-18). This establishes a positive feedback loop that further enhances NET formation and perpetuates inflammation (37–39). Bruton’s tyrosine kinase has been reported to exert a regulatory role in this process (40). Beyond direct signaling effects, NETs also amplify inflammation by orchestrating intercellular immune interactions. Their structural components, along with associated cytokines and chemokines, function as signaling platforms that promote the activation and recruitment of neutrophils, macrophages, B cells, and other immune populations (41). In addition, NETs activate multiple signaling pathways, including cGAS-STING, TLR9, and transforming growth factor-β (TGF-β), leading to robust macrophage activation and polarization toward a pro-inflammatory phenotype. This is accompanied by increased secretion of IL-1β, IL-18, and B cell-activating factor, further amplifying local inflammatory responses (28, 42–44). In AS, NETs have also been shown to inhibit macrophage autophagy via the EGFR-Beclin-1 signaling pathway and modulate inflammasome activation, thereby accelerating disease progression (45). Collectively, NETs integrate innate immune sensing, inflammasome activation, and immune cell crosstalk through their structural components and diverse signaling pathways, thereby sustaining inflammatory amplification in CVD (**Figure 2**).

## 4 Inflammation mediated by NETs in CVD

### 4.1 Atherosclerosis

AS is a chronic inflammatory disease characterized by endothelial dysfunction, immune inflammatory activation, and vascular smooth muscle cell (VSMC) proliferation. NETs contribute to disease progression by activating innate immune responses, amplifying inflammatory signaling, and promoting endothelial injury. Several NET associated components, including DNA, NE, histones, MPO, and serine proteases, have been implicated in the inflammatory processes underlying AS (36, 46). For example, proteinase 3 and CG can activate proinflammatory cytokines such as tumor necrosis factor α (TNF-α), IL-18, and IL-1β, thereby initiating inflammatory responses (47). Experimental studies have further shown that NE activates the NLRP3 inflammasome through the TLR4/MyD88/IRAK1/TRAF6/NF-κB signaling pathway (48), promoting proinflammatory responses in VSMCs (49). In addition, NETs increase the expression of vascular cell adhesion molecule 1 (VCAM-1), intercellular adhesion molecule 1 (ICAM-1), vascular endothelial growth factor A, and IL-6 in human umbilical vein endothelial cells, promoting inflammatory cell adhesion, intraplaque neovascularization, and endothelial cell death (50). These effects have been linked to the development of unstable atherosclerotic plaques (51). Moreover, NET-mediated activation of the NLRP3 inflammasome and enhancement of IL-8 signaling further aggravate endothelial inflammation, whereas IL-1α and CG induced tissue factor expression promotes endothelial dysfunction and a prothrombotic state (52, 53). The proinflammatory, endothelial disruptive, and prothrombotic effects of NETs act in concert to drive plaque initiation, progression, and destabilization, supporting their important role in linking vascular inflammation to adverse cardiovascular outcomes.

NETs also participate in several atherosclerosis specific processes associated with lipid accumulation and plaque microenvironment remodeling. Animal studies have shown that hypercholesterolemia impairs endoplasmic reticulum stress dependent DNase activity, resulting in defective NET clearance and persistent NET accumulation (54, 55). Meanwhile, cholesterol crystal uptake by VSMCs promotes IL-33 release and ROS dependent NET formation, which recruits additional immune cells and amplifies local inflammatory responses (56, 57). Under metabolic conditions such as diabetes mellitus, NETs induce macrophage metabolic reprogramming and inflammasome activation, acting in concert with cholesterol crystals to drive the sustained release of proinflammatory cytokines and perpetuate inflammatory responses (58). Recent evidence has further demonstrated that macrophage-derived exosomal miR-146a-5p regulates NET formation through the PNKP/DDOST/JAGN1 axis, thereby exacerbating vascular inflammation. In experimental models, inhibition of this pathway reduced NET formation and attenuated plaque progression (59). NETs have also been shown to promote Th17 cell differentiation and IL-17 production, further enhancing immune cell infiltration and systemic inflammation (60). Collectively, these findings suggest that, in addition to promoting inflammatory signaling and endothelial dysfunction, NETs contribute to plaque microenvironment remodeling through impaired clearance, cholesterol crystal induced activation, and interactions between macrophages and VSMCs, thereby facilitating plaque progression and instability.

### 4.2 Acute coronary syndromes and myocardial ischemia-reperfusion injury

During acute coronary syndrome (ACS) and myocardial I/R injury, neutrophils are among the earliest immune cells recruited to the injured myocardium (61). Increasing evidence indicates that NETs are key mediators of inflammatory responses following MI. Similar to their role in AS, NETs contribute to myocardial injury by activating inflammatory pathways, recruiting immune cells, and modulating macrophage function. NET related hub genes, including IL1B, MMP9, CXCL2, and ICAM1, have been identified in clinical studies, supporting the involvement of NETs in post infarction inflammation and tissue injury (62). Experimental studies have further shown that PAD4, a critical regulator of NET formation, promotes NET release and the production of proinflammatory mediators, including IL-1, IL-6, and TNF-α. Inhibition of PAD4 attenuates inflammatory responses and cardiomyocyte apoptosis, thereby reducing myocardial injury (63). Consistent with these observations, clinical studies have demonstrated associations among IL-6 receptor signaling, PAD4 expression, and NET formation, further supporting PAD4 as a potential therapeutic target in MI (64). Beyond their role in amplifying inflammation, NETs have been implicated in microvascular injury and reperfusion related complications. In patients with ST segment elevation myocardial infarction (STEMI), NETs and extracellular chromatin have been shown to accumulate at culprit lesion sites and are associated with microvascular obstruction (MVO), elevated C-reactive protein (CRP) and IL-6 levels, and adverse clinical outcomes (65). In murine models of myocardial I/R injury, the TRAIL-DR5 signaling pathway has emerged as an important regulator of NET mediated inflammatory responses. Activation of this pathway enhances neutrophil infiltration through upregulation of inflammatory molecules, including ICAM-1, Cxcl15, and Itgam, whereas its inhibition mitigates inflammatory injury and reduces myocardial damage (66). Collectively, these findings suggest that NETs not only intensify post infarction inflammation but also contribute to microvascular dysfunction and reperfusion injury, underscoring their distinctive role in the acute pathophysiology of ACS.

Notably, the role of NETs following MI extends beyond the acute injury phase and may influence both the resolution of inflammation and subsequent tissue repair. Experimental studies have shown that NETs regulate macrophage recruitment and phenotypic transition and, together with other immune cells, affect myocardial necrosis, scar formation, and cardiac remodeling (67). Mice with impaired NET formation exhibit reduced upregulation of M2 associated genes and heightened acute inflammatory responses after myocardial infarction (68), suggesting that NETs may contribute to the resolution phase of post infarction inflammation. Mechanistically, NETs have been reported to promote the polarization of proinflammatory Mertk^+^MHC II^low/int macrophages through Toll-like receptor 9 signaling, thereby influencing the balance between tissue injury and repair (69). In addition, PAD4 and APOE may indirectly enhance NET formation through their effects on macrophage polarization, further modulating local inflammatory activity (67). Interestingly, some studies have reported aggravated myocardial I/R injury following PAD4 deficiency (70), indicating that the effects of NETs during cardiac injury and repair may vary according to the pathological context. NETs also increase the expression of MCP-1 and ICAM-1, promote monocyte recruitment, and facilitate differentiation toward profibrotic phenotypes, thereby contributing to both inflammatory amplification and tissue remodeling (71). Collectively, these findings indicate that NETs participate in ACS and myocardial I/R injury through multiple interconnected mechanisms involving inflammatory signaling, immune cell recruitment, and cellular polarization. Dysregulated NET activity not only contributes to coronary microvascular dysfunction and myocardial injury after reperfusion but also influences subsequent cardiac repair and remodeling.

### 4.3 Heart failure

HF represents the final common pathway of many CVD and is characterized by persistent low grade inflammation, adverse cardiac remodeling, and progressive decline of cardiac function. Clinical studies have demonstrated increased neutrophil activation and enhanced NET formation in patients with HF (72, 73), which correlate positively with circulating inflammatory mediators, including IL-6, IL-8, and CRP, as well as the severity of cardiac dysfunction (74). These observations suggest a link between NET associated inflammation and functional impairment in HF (34, 74). In addition, experimental studies have shown that platelet activating factor (PAF) released from injured cardiomyocytes induces NETosis, thereby promoting NET formation and amplifying local inflammatory responses. Pharmacological inhibition of PAF signaling or NET formation attenuates inflammation and tissue injury, supporting a role for NETs as mediators linking myocardial damage to sustained inflammatory activation (65).

A distinctive feature of NET involvement in HF is its close association with myocardial fibrosis and structural remodeling. Experimental studies have demonstrated that NETs not only promote the differentiation of monocytes into fibroblast like cells but also directly activate cardiac fibroblasts, contributing to extracellular matrix deposition and myocardial fibrosis (71, 75). In diabetic cardiorenal disease, neutrophils enhance NET and IL-1β release through activation of the PAD4 inflammasome axis, accompanied by aggravated cardiac and renal fibrosis and increased levels of the endothelial activation marker von Willebrand factor (76). These findings suggest that NETs may contribute to pathological crosstalk between the heart and kidney. In HF with preserved ejection fraction, increased NET formation has been observed in both the circulation and myocardial tissue. Mechanistic studies indicate that NETs promote macrophage infiltration, myocardial fibrosis, and diastolic dysfunction through high mobility group box 1 (HMGB1) associated inflammatory signaling. Inhibition of NET formation, blockade of HMGB1 signaling, or treatment with sodium glucose cotransporter 2 inhibitors such as empagliflozin attenuates inflammation and improves cardiac function (77). Similarly, in hypertensive heart disease, NETs induce oxidative stress and activate NF-κB signaling in cardiomyocytes, promoting ferroptosis and inflammatory injury. NETs also upregulate profibrotic mediators, including fibronectin, collagen, and α-smooth muscle actin, thereby accelerating myocardial fibrosis and functional decline (78). Current evidence identifies NETs as important mediators linking inflammation, fibrosis, and organ remodeling in HF. Beyond sustaining chronic immune activation, NETs appear to contribute directly to myocardial fibrogenesis, adverse cardiac remodeling, and cardiorenal interactions. Further elucidation of NET driven mechanisms across different HF phenotypes may help establish their potential as therapeutic targets and biomarkers for HF.

### 4.4 Hypertension

Hypertension is characterized not only by hemodynamic abnormalities but also by persistent innate immune activation and chronic low grade inflammation. NETs contribute to blood pressure elevation and target organ injury through their proinflammatory, prothrombotic, and cytotoxic properties. Recent clinical studies have demonstrated enhanced NETosis and reduced circulating DNase I levels in patients with hypertension, suggesting impaired NET clearance under hypertensive conditions. NET accumulation resulting from defective degradation may sustain local and systemic immune activation through innate immune pathways such as Toll like receptor 9, which can be activated by hypomethylated CpG DNA released from NETs (79, 80). Current research has primarily focused on the interactions between NETs, the renin-angiotensin-aldosterone system, and vascular dysfunction. Angiotensin II has been identified as a potent inducer of NET formation (81). Mechanistic studies further indicate that citrullinated histone H3 (CitH3), a hallmark product of NETosis, promotes the expression of cytokines and profibrotic mediators, including IL-6, TGF-β, and TNF-α. Conversely, PAD4 deficiency markedly attenuates Ang II induced hypertension and reduces the infiltration of neutrophils, dendritic cells, and CD8^+^ T cells into target organs (82). These findings support the concept that the Ang II-PAD4-NET axis represents an important pathway linking immune activation to blood pressure regulation. In addition to inflammatory responses, NETs also contribute to vascular remodeling and thrombotic susceptibility in hypertension. Clinical studies have reported associations between elevated NET levels and hypercoagulability in patients with severe essential hypertension (83). Experimental evidence further demonstrates that NETs promote thrombin generation and fibrin formation while inducing a procoagulant phenotype in endothelial cells, thereby enhancing thrombogenicity (84). In parallel, NETs stimulate vascular smooth muscle cell proliferation through activation of the Akt/CDKN1b/TK1 signaling pathway. These proliferative effects may be further propagated through TK1-enriched extracellular vesicles, contributing to pathological vascular remodeling (85). Collectively, current evidence indicates that NETs participate in several key pathological processes underlying hypertension, including immune activation, vascular remodeling, and coagulation abnormalities. By integrating inflammatory, thrombotic, and vascular remodeling pathways, NETs may contribute not only to blood pressure elevation but also to hypertensive target organ damage.

### 4.5 Atrial fibrillation

Atrial fibrillation (AF) is the most common sustained cardiac arrhythmia and is characterized by chronic inflammation and progressive atrial remodeling. Clinical studies have demonstrated that elevated levels of NET associated biomarkers correlate with left atrial enlargement and reduced left ventricular ejection fraction (86). Increased NET activity has also been linked to a higher risk of AF related stroke (87), suggesting a role for NETs in both atrial remodeling and thromboembolic. S100A8/A9, a major NET component and member of the damage associated molecular pattern family, is released during NET formation and can amplify local inflammatory responses (88). In a chronic mild stress mouse model, increased atrial expression of MPO, CitH3, and S100A8/A9 activated the TLR4/NLRP3 signaling pathway, promoted NET formation and inflammatory cell infiltration, and contributed to atrial remodeling (89). These findings indicate a close relationship between NET mediated inflammation and atrial structural changes. NETs may also contribute to the development of an inflammatory atrial environment. Epicardial adipose tissue (EAT), an important source of local inflammatory mediators, is enriched with myeloperoxidase containing secretory products. Neutrophils residing within EAT release substantial amounts of NETs, with the highest NET burden observed in patients with persistent AF. NETs stimulate the expression of extracellular matrix related genes in atrial fibroblasts, thereby promoting atrial fibrosis and facilitating both the initiation and maintenance of AF (90, 91). Clinical studies further indicate that elevated circulating NE levels are independently associated with all cause mortality, cardiovascular mortality, and other adverse cardiovascular events in anticoagulated patients with AF. Increased NE activity has also been linked to dysregulation of the anti-inflammatory microRNA miR-146a, which may facilitate persistent inflammatory activation and continued NET formation. These findings suggest that NET driven inflammation may contribute to unfavorable clinical outcomes in AF (92). Overall, current evidence indicates that NETs participate in multiple pathological processes underlying AF, including inflammatory responses, fibroblast activation, atrial fibrosis, and structural remodeling. By fostering a profibrotic and proinflammatory atrial milieu, NETs may contribute not only to AF initiation and persistence but also to its thromboembolic and cardiovascular complications.

### 4.6 Myocarditis

Myocarditis is characterized by immune mediated myocardial injury and inflammatory cell infiltration. NETs contribute to myocardial inflammation by amplifying inflammatory signaling and promoting immune cell recruitment. Through the release of extracellular DNA and granule proteins, including MPO and NE, NETs stimulate the production of proinflammatory mediators such as IL-8 and activate inflammasome related pathways. These processes promote monocyte and macrophage activation as well as functional reprogramming, resulting in enhanced macrophage infiltration and more severe myocardial injury (93). The effects of NETs differ among distinct forms of myocarditis. In doxorubicin induced cardiotoxicity, NETs promote the release of IL-18 from macrophages, which subsequently stimulates interferon γ production by T cells and amplifies inflammatory responses. NETs also reduce connexin 43 expression and impair cardiac electrical conduction, contributing to arrhythmias and aggravating myocardial injury (94). In coxsackievirus B3(CVB3) induced viral myocarditis, NET formation is more closely associated with virus driven immune responses. CVB3 activates neutrophils through TLR8 dependent signaling, leading to increased NET release. NET related inflammatory mediators, including IL-1β, TNF-α, CCL2, and CXCL10, further promote monocyte recruitment and differentiation toward Ly6C^High inflammatory macrophages, leading to persistent myocardial inflammation and tissue damage (95). In addition, midkine (MK) has emerged as an important regulator of NET formation. Through interaction with its receptor, low-density lipoprotein receptor related protein 1, MK promotes neutrophil recruitment and NET generation, further enhancing inflammatory activity within the myocardium (96). Collectively, current evidence indicates that NETs contribute to myocarditis through shared mechanisms involving inflammatory amplification and immune cell recruitment. These findings underscore the multifaceted role of NETs in shaping myocardial immune responses and disease progression across different forms of myocarditis.

## 5 Clinical biomarkers

NET mediated immune responses, inflammation, and thrombosis play a central role in the pathogenesis of CVDs. Accordingly, NET related molecules have gradually emerged as novel biomarkers for risk stratification and disease evaluation. In recent years, accumulating evidence has shown that NETs and their associated markers, such as NE, MPO, dsDNA, and CitH3, are significantly elevated in various cardiovascular conditions, including AS, MI, HF, and AF (97–99), as summarized in **Table 1**. These markers have been associated with key pathological features, such as infarct size, microvascular obstruction, left ventricular remodeling, and reduced ejection fraction. Furthermore, several studies suggest that integrating NET related components with conventional clinical risk factors may improve diagnostic and prognostic performance by enabling multidimensional risk stratification. This approach may facilitate the identification of high risk patient populations and support the use of NET associated biomarkers as independent predictors of MACE, all cause mortality, and long term adverse outcomes (97, 116). Therefore, dynamic monitoring of NET related markers may provide additional value in predicting both short term and long term clinical outcomes, highlighting their potential clinical utility.

**Table 1:** NET related biomarkers for the diagnosis and evaluation of cardiovascular diseases.

NETs biomarkers	Study population	Study design	Sample source	Main findings	Reference
MPO-DNA	Primary hypertension	Cross sectional study	Human peripheral blood	MPO-DNA levels were positively associated with blood pressure and independently correlated with carotid pulse wave velocity.	(100)
NETs	STEMI	Prospective cohort study	Human coronary thrombus	Increased NET formation was associated with elevated high-sensitivity C-reactive protein levels.	(101)
MPO-DNA, NE-DNA, CitH3	MI	Case control study	Human peripheral serum	A composite biomarker score based on NET-related markers predicted the occurrence of major adverse cardiovascular events.	(102)
dsDNA	NSTEMI	Prospective cohort study	Human peripheral blood	dsDNA independently predicted the composite outcome of death, myocardial infarction, or hospitalization for heart failure.	(97)
CitH3	Heart failure	Cross sectional study	Human peripheral blood	CitH3 levels were inversely correlated with left ventricular ejection fraction.	(98)
NETs	Atrial fibrillation	Cross sectional study	Peripheral blood	NET-related markers were positively correlated with spontaneous echo contrast (SEC) scores and improved the predictive value of the CHA_2_DS_2_-VASc score for thrombotic risk.	(99)
MPO-DNA complex	STEMI	Prospective cohort study	Peripheral serum	Predicted adverse left ventricular remodeling after PCI in patients with STEMI.	(103)
NE, MPO, dsDNA	MI	Prospective cohort study	Peripheral blood and coronary thrombus	Predicted adverse cardiovascular events.	(104, 105)
H3Cit-DNA	STEMI	Prospective clinical cohort study	Peripheral blood	Predicted short-term adverse cardiovascular events.	(106)
NETs	STEMI	Prospective cohort study	Coronary thrombus	Significantly associated with major adverse cardiac events within 30 days after PCI.	(107)
dsDNA	STEMI	Cross sectional study	Peripheral blood	dsDNA levels were positively correlated with cardiac troponin T concentrations and showed potential prognostic value.	(35)
dsDNA	STEMI	Prospective cohort study	Peripheral blood	Closely associated with microvascular obstruction and reduced ejection fraction.	(65)
NETs	STEMI	Cross sectional study	Coronary thrombus	Associated with delayed reperfusion, stress hyperglycemia, impaired cardiac function, and distal embolization risk.	(108)
H3Cit-DNA	STEMI	Prospective cohort study	Peripheral blood	Predicted short-term mortality and major adverse cardiovascular events.	(109)
dsDNA	STEMI	Prospective cohort study	Peripheral serum	Associated with larger infarct size, adverse left ventricular remodeling, and increased risk of adverse clinical outcomes.	(110)
dsDNA	STEMI	Prospective cohort study	Peripheral serum	Significantly associated with increased all-cause mortality in STEMI patients.	(111)
NETs	STEMI		Coronary thrombus and serum	Elevated coronary NET burden was associated with larger infarct size and impaired ST-segment resolution.	(112)
NETs and NE	ACS	Cross sectional study	Peripheral blood	NET levels were elevated in serum and neutrophils of myocardial infarction patients, accompanied by increased NE expression and activity, and positively correlated with IL-6 levels.	(113)
NETs	Idiopathic dilated cardiomyopathy with heart failure	Prospective cohort study	Myocardial tissue and peripheral blood	Associated with reduced LVEF and elevated B-type natriuretic peptide levels, and independently predicted adverse cardiac events.	(114)
dsDNA and MPO-DNA	STEMI complicated with acute heart failure	Retrospective longitudinal study	Peripheral blood	Associated with myocardial function and IL-8 levels.	(115)

NETs, neutrophil extracellular traps; MPO-DNA, myeloperoxidase-DNA complex; NE-DNA, neutrophil elastase-DNA complex; CitH3, citrullinated histone H3; dsDNA, double-stranded DNA; NE, neutrophil elastase; H3Cit-DNA, citrullinated histone H3-DNA complex; STEMI, ST-segment elevation myocardial infarction; NSTEMI, non-ST-segment elevation myocardial infarction; MACE, major adverse cardiovascular events; LVEF, left ventricular ejection fraction; SEC, spontaneous echo contrast; CHA_2_DS_2_-VASc, congestive heart failure, hypertension, age ≥75 years, diabetes mellitus, stroke/transient ischemic attack/thromboembolism, vascular disease, age 65–74 years, and sex category; PCI, percutaneous coronary intervention; cTnT, cardiac troponin T; IL-6, interleukin-6; IL-8, interleukin-8; NCT, National Clinical Trial identifier.

Although substantial evidence supports the potential value of NET related biomarkers in the diagnosis, risk stratification, and prognostic evaluation of cardiovascular diseases, several factors continue to limit their clinical application. One of the most important challenges is the lack of standardized methods for NET quantification, which affects analytical sensitivity, specificity, and reproducibility across studies (117, 118). Commonly used biomarkers include circulating cfDNA, MPO DNA complexes, CitH3, and NE, however, these markers differ considerably in their specificity for NET formation (116). For example, cfDNA may originate not only from NET release but also from apoptosis, necrosis, and other forms of cell death (119). In contrast, measurements of MPO DNA complexes and CitH3 can be influenced by assay platforms, antibody sources, and experimental conditions (120–122). These methodological inconsistencies likely contribute to the discrepancies observed across studies. The interpretation of NET related biomarkers is further complicated by preanalytical variables. Sample type, processing time, centrifugation protocols, storage conditions, and repeated freeze thaw cycles can all influence the stability and quantification of NET derived components. Consequently, these factors may introduce substantial interstudy heterogeneity and compromise the comparability of results. Comorbid conditions represent another important source of confounding. Diseases such as infection, diabetes mellitus, and cancer can promote NET formation or impair NET clearance pathways (123–125), thereby influencing circulating NET levels independently of cardiovascular disease. As a result, elevated NET related biomarkers may not exclusively reflect cardiovascular pathology, potentially limiting their disease specificity and clinical interpretability. The absence of universally accepted reference ranges and diagnostic thresholds also remains a major barrier. Differences in study populations, disease stages, analytical techniques, and outcome definitions have resulted in considerable variation in reported cutoff values (97–99, 102). Without standardized thresholds, direct comparisons among studies remain challenging, hindering the integration of NET related biomarkers into routine clinical practice. Further progress in this field will require greater methodological standardization, validation in large multicenter cohorts, and the establishment of clinically meaningful reference intervals and diagnostic thresholds. Addressing these challenges will be essential for translating NET related biomarkers from experimental research tools into reliable indicators for CVD diagnosis, risk assessment, and therapeutic decision making.

## 6 Targeted therapies against NETs

As described above, NET mediated inflammatory responses are involved in the progression of multiple CVD. Increasing evidence indicates that targeting NETs holds considerable therapeutic potential in CVD. Current strategies include the inhibition of NET formation, such as calcineurin inhibitors, ROS scavengers, PAD inhibitors, and DNase-based approaches, which represent potential therapeutic options (126). Additional strategies involve promoting NET clearance and modulating upstream inflammatory signaling pathways, thereby attenuating inflammatory responses, reducing infarct size, and improving cardiac function. Moreover, mesenchymal stem cell-derived exosomes, as well as several pharmacological agents such as colchicine and tocilizumab, have been shown to exert cardioprotective effects, at least in part, through the regulation of NET formation. In parallel, nanoparticle based drug delivery systems have emerged as a promising approach by improving drug stability, enabling controlled release, and enhancing bioavailability (127, 128), representing an additional therapeutic strategy. In addition, bioactive components derived from natural compounds have also been reported to modulate NET related pathways, as summarized in **Table 2**.

**Table 2:** Advances in NET targeted therapeutic strategies for cardiovascular diseases.

Intervention strategy	Representative agent	Target/Mechanism	Evidence source	Major effects	References
Stem cell-based therapy	MSC-Exo (miR-199)	Suppression of NET formation and NLRP3 inflammasome activation	Animal and *in vitro* studies	Attenuated myocardial I/R injury, reduced infarct size, alleviated microvascular obstruction, and improved cardiac function	(129)
Anti-inflammatory monoclonal antibody	Tocilizumab	IL-6 signaling pathway	Human studies	Improved myocardial salvage index, reduced infarct size, and mitigated microvascular obstruction	(130)
Microtubule inhibitor	Colchicine	Inhibition of PAD4 and cytoskeletal modulation	Animal studies	Reduced perioperative myocardial injury	(131)
Natural compound combination	Ferulic acid (FA) + Rb1	Inhibition of platelet-derived HMGB1 release	Animal and *in vitro* studies	Improved cardiac function and reduced no-reflow area and microthrombus burden	(132)
Anti-inflammatory agent	Colchicine	Suppression of NET formation	Human studies	Inhibited post-PCI inflammation and microthrombus formation	(133)
Leukotriene receptor antagonist	Pranlukast	Inhibition of NETosis	Animal studies	Reduced myocardial infarct size	(103)
Complement pathway inhibitor	13-Me-PLT	Blockade of the C5a-C5aR1 axis	Animal and *in vitro* studies	Improved cardiac function and suppressed inflammatory responses	(134)
Anti-NET antibody	hCitH3-mAb	Targeting CitH3	Animal studies	Reduced infarct size, inhibited inflammatory cytokine elevation, and preserved mitochondrial function	(135)
Metabolic modulation	FMN	Inhibition of platelet activation and CD36 signaling pathway	Animal and *in vitro* studies	Attenuated thrombosis and inflammation in myocardial ischemia/reperfusion injury	(136)

MSC-Exo, mesenchymal stem cell-derived exosomes; miR-199, microRNA-199; NETs, neutrophil extracellular traps; NLRP3, NOD-like receptor family pyrin domain containing 3; I/R, ischemia/reperfusion; IL-6, interleukin-6; MSI, myocardial salvage index; PAD4, peptidyl arginine deiminase 4; FA, ferulic acid; HMGB1, high mobility group box 1; PCI, percutaneous coronary intervention; NETosis, neutrophil extracellular trap formation; C5aR1, complement component 5a receptor 1; hCitH3-mAb, humanized citrullinated histone H3 monoclonal antibody; CitH3, citrullinated histone H3; FMN, formononetin; CD36, cluster of differentiation 36.

Although therapies targeting NETs have demonstrated therapeutic potential in various cardiovascular diseases, several challenges continue to hinder their clinical application. NETs are a key component of innate immunity and play important roles in pathogen containment, microbial clearance, and tissue repair. Accordingly, excessive inhibition of NET formation may compromise host defense mechanisms (137) and increase susceptibility to infection, particularly in individuals with immune dysfunction, advanced age, diabetes mellitus, chronic kidney disease, or other conditions associated with impaired immunity. Current evidence also indicates that the biological effects of NETs vary according to the pathological context (138). Persistent or excessive NET formation can promote chronic inflammation and tissue injury, whereas transient NET generation may facilitate the clearance of necrotic debris, promote inflammatory resolution, and contribute to tissue repair. Therefore, complete inhibition of NET formation may interfere with inflammatory resolution and alter macrophage polarization during infarct healing (68). These findings suggest that NETs cannot be regarded solely as pathogenic factors, as they may also exert protective effects that contribute to cardiovascular homeostasis. Distinguishing pathological NET activity from physiological or reparative NET responses thus remains a critical challenge in the development of NET targeted therapies. Another issue is the heterogeneity of NET formation pathways. NETs can be generated through both PAD4 dependent and PAD4 independent mechanisms and interact extensively with immune and nonimmune cell populations. Consequently, broad inhibition of NET formation may lead to unintended systemic effects while further increasing the risk of infection. By contrast, therapeutic strategies targeting disease specific signaling pathways or key molecular mediators may provide greater selectivity and potentially improved safety profiles (118). The central therapeutic challenge is therefore not simply whether NET formation should be inhibited, but how pathological NET activity can be selectively modulated while preserving its physiological functions. Future therapeutic strategies may benefit from patient stratification, biomarker guided treatment, and interventions tailored to different stages of disease. Such strategies may improve the balance between efficacy and safety and facilitate the clinical translation of NET targeted therapies in cardiovascular diseases.

## 7 Discussion

Despite growing evidence implicating NET mediated inflammation in the pathogenesis of AS, MI, HF, and myocarditis, several fundamental questions remain unresolved. The most important is whether NETs are true drivers of cardiovascular disease progression or primarily reflect ongoing inflammatory activity. Although elevated NET levels are consistently associated with disease severity, adverse cardiovascular events, and poor prognosis, current evidence remains largely associative. Establishing causality will require integrated approaches combining genetic manipulation, disease specific experimental models, and longitudinal clinical studies. In addition, the biological effects of NETs appear to be highly context dependent. Excessive NET formation can amplify inflammation, thrombosis, and tissue injury, whereas controlled NET activity may contribute to debris clearance and tissue repair. Similar dual effects may exist across different stages of atherosclerosis, myocardial remodeling, and infarct healing, suggesting that NETs function as dynamic regulators rather than uniformly pathogenic mediators. Defining the temporal and disease specific determinants of NET activity therefore remains essential for understanding their role in cardiovascular pathology.

From a translational perspective, substantial challenges continue to limit the clinical application of NET related biomarkers and NET targeted therapies. The lack of standardized methods for NET detection and quantification, together with variability in biomarker selection and cutoff definitions, restricts their utility in diagnosis, risk stratification, and prognostic assessment. Moreover, because NETs play important roles in host defense and tissue homeostasis, indiscriminate inhibition may compromise physiological immune functions. Future efforts should therefore focus on identifying disease specific regulatory pathways, developing selective therapeutic strategies, and defining the patient populations and therapeutic windows most likely to benefit from NET targeted interventions. Addressing these challenges will be critical for translating mechanistic insights into clinically meaningful applications in cardiovascular medicine.

## Glossary

CVD: cardiovascular disease
NETs: neutrophil extracellular traps
MACE: major adverse cardiovascular events
NE: neutrophil elastase
MPO: myeloperoxidase
DAMPs: damage-associated molecular patterns
I/R: ischemia-reperfusion
ROS: reactive oxygen species
PAD4: peptidyl arginine deiminase 4
LPS: lipopolysaccharide
PKC: protein kinase C
GSDMD: gasdermin D
NOX: NADPH oxidase
mtROS: mitochondrial reactive oxygen species
dsDNA: double-stranded DNA
TLR4: Toll-like receptor 4
IL-1β: interleukin-1β
TGF-β: transforming growth factor-β
VSMC: vascular smooth muscle cell
CG: cathepsin G
VCAM-1: vascular cell adhesion molecule 1
ICAM-1: intercellular adhesion molecule 1
ACS: acute coronary syndromes
STEMI: ST-segment elevation myocardial infarction
CRP: C-reactive protein
HF: heart failure
PAF: platelet activating factor
HMGB1: high-mobility group box 1
CitH3: citrullinated histone H3
AF: atrial fibrillation
AS: atherosclerosis
MI: myocardial infarction
TNF-α: tumor necrosis factor α.
